# Moisture Prediction of Transformer Oil-Immersed Polymer Insulation by Applying a Support Vector Machine Combined with a Genetic Algorithm

**DOI:** 10.3390/polym12071579

**Published:** 2020-07-16

**Authors:** Yiyi Zhang, Jiaxi Li, Xianhao Fan, Jiefeng Liu, Heng Zhang

**Affiliations:** Guangxi Key Laboratory of Power System Optimization and Energy Technology, Guangxi University, Nanning 530004, Guangxi, China; yiyizhang@gxu.edu.cn (Y.Z.); lijiaxi1995@st.gxu.edu.cn (J.L.); xianhao_fan@163.com (X.F.); hengzhang_gxu@163.com (H.Z.)

**Keywords:** transformer oil-immersed polymers insulation, frequency domain spectroscopy (FDS), genetic algorithm (GA), support vector machine (SVM), moisture prediction

## Abstract

The support vector machine (SVM) combined with the genetic algorithm (GA) has been utilized for the fault diagnosis of transformers since its high accuracy. In addition to the fault diagnosis, the condition assessment of transformer oil-immersed insulation conveys the crucial engineering significance as well. However, the approaches for getting GA-SVM used to the moisture prediction of oil-immersed insulation have been rarely reported. In view of this issue, this paper pioneers the application of GA-SVM and frequency domain spectroscopy (FDS) to realize the moisture prediction of transformer oil-immersed insulation. In the present work, a method of constructing a GA-SVM multi-classifier for moisture diagnosis based on the fitting analysis model is firstly reported. Then, the feasibility and reliability of the reported method are proved by employing the laboratory and field test experiments. The experimental results indicate that the reported prediction model might be serviced as a potential tool for the moisture prediction of transformer oil-immersed polymer insulation.

## 1. Introduction

Power transformers perform a vital task of transforming electrical energy in power systems, and the condition evaluation of its internal oil/paper system has attracted wide attention [1,2,3,4,5,6]. In recent years, a prevailing method of moisture determination is the so-called frequency domain spectroscopy (FDS) [7,8,9,10] technique, which is also called dielectric frequency response (DFR) technique. The condition assessment of transformer oil-immersed polymer insulation based on FDS could be realized by the following steps: Firstly, extracting feature parameters that could reflect the insulation conditions of oil-immersed insulation from the measured FDS data. Then, establishing the quantitative relationship between these feature parameters and insulating states, and, finally, the insulating status of the oil-immersed insulation can be evaluated by the quantity relationship. Existing studies [11,12] indicated that the insulation conditions of power transformers could be affected by various factors, such as moisture, thermal, oxygen, and acids.

Moreover, for every 0.5% increase in moisture, the life of oil/paper insulation will be shortened greatly [13], and the increasing moisture reduces the breakdown voltage, which leads to equipment accidents [14]. Therefore, the moisture diagnosis is of great significance to the transformer operating condition, and such the topic is therefore treated as the investigating theme in this work. Review existing investigation, the approaches for oil-immersed insulating condition prediction based on dielectric response spectroscopy can be divide into two types. One is based on extracting the feature parameters from the dielectric response curves [11,15,16,17]. The other is realized by establishing the equivalent circuit model to extract the feature parameters of dielectric response property [18,19]. The above methods could extract the feature parameters, reflecting the moisture inside oil-immersed insulation.

As for the establishment of the quantitative relationship between feature parameters and the insulating states, the traditional methods (such as fitting analysis [12]) achieve it by using the small number of samples, which were faced with the limitation of simple classification (diagnosis) basis, no generalization ability, and difficulty in extrapolation. Fortunately, the support vector machine (SVM) combined with the genetic algorithm (GA) can be utilized to overcome these issues. The GA-SVM is a powerful tool for solving the problem with nonlinearity and high dimension [20], which has been employed to deal with the problem of transformer fault diagnosis [20,21,22,23]. While the GA-SVM cannot be applied to the moisture diagnosis directly since the training of the GA-SVM moisture prediction model needs plenty of training samples (oil-immersed pressboards). However, it is a challenging task to prepare a great number of oil-immersed pressboards with different expected moisture content (*mc%*). Moreover, the feasibility verification after model construction is a problem to be overcome.

In consideration of these issues, a series of approaches is proposed in this article. Firstly, a fitting analysis model is put forward, which is aimed to generate fitting fingerprints serving as training samples. Then, the fitting fingerprints are utilized to train the moisture prediction model based on GA-SVM. Finally, the verification test in lab and field conditions is performed and the feasibility and accuracy of the GA-SVM model are proved. Therefore, the experimental result indicates that the GA-SVM model proposed in this article might provide a novel ideal for the moisture prediction of the oil-immersed polymers insulation in transformers.

## 2. Sample Preparation and Frequency Response Test

The aging effect on FDS data must be taken into consideration when using FDS to evaluate the moisture content of oil-immersed insulation [24,25]. In that case, the oil-immersed polymer (cellulose) pressboards with five different aging/damp statuses have been prepared in this work, where the details of the cellulosic pressboards and insulating oil are displayed in Table 1.

The cellulosic pressboards and insulating oil are firstly dried in the vacuum tank at 105 °C/50 Pa for 48 h. Then, they are placed into the vacuum tank at 60 °C/50Pa for 48 h to complete the vacuum impregnation. In that way, the oil-immersed pressboards are prepared. Afterward, the prepared oil-immersed pressboards are divided into five groups and placed into five different aging cans to perform an accelerating aging experiment at 150 °C for 0 day, 1 day, 3 days, 7 days, and 14 days, respectively. Finally, the oil-immersed pressboards with initial *mc%* (*a%*) are placed in a precision electronic balance and their quality (*m*) is recorded; the natural moisture absorption is later performed to obtain the expected moisture by controlling the quality. Provided that the measured value of the balance reaches m * (1 + *b%*)/(1 + *a%*), the moisture content of the pressboard is regarded as *b%*. In this work, the expected moisture content is 1%, 2%, 3%, and 4%, respectively. Above all, the oil-immersed pressboards with various aging conditions and moisture content are prepared.

The prepared oil-immersed pressboards are put into a three-electrode device to finish the frequency response test with the help of a dielectric response analyzer, i.e. DIRANA tester. The three-electrode device is filled with dried and degassed insulating oil and the pressboard is immersed in the insulating oil to simulate the off-line measurement status (vacuum and oil immersion) of the transformer main insulation.

**Figure 1:**
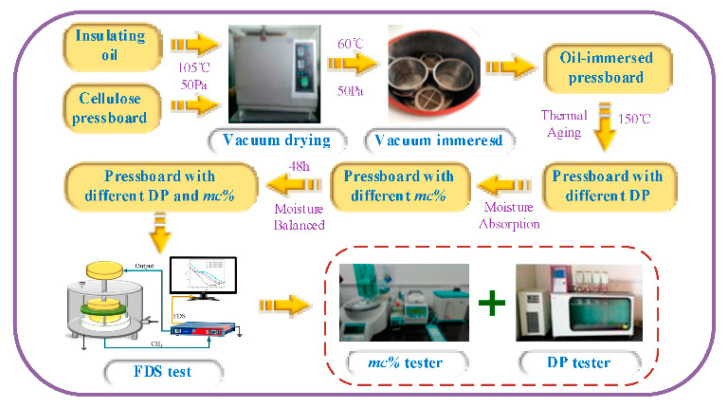
Schedule of sample preparation and insulting information testing.

Afterward, the moisture is tested by the Metrohm Coulomb Karl Moisture Tester (as shown in Figure 1) and based on International Electrotechnical Commission standard, IEC 60814, the degree of polymerization (*DP*) is tested by the Automatic Viscosity Tester (as shown in Figure 1) and based on IEC 60450-2007, respectively. The experiment schedule is shown in Figure 1. Besides, according to the preset moisture values, the moisture content of the oil-immersed pressboard can be divided into eight different levels. The classification of moisture content is shown in Table 2.

## 3. Acquisition of Fitting Fingerprints

### 3.1. Analysis of FDS Curves

**Figure 2:**
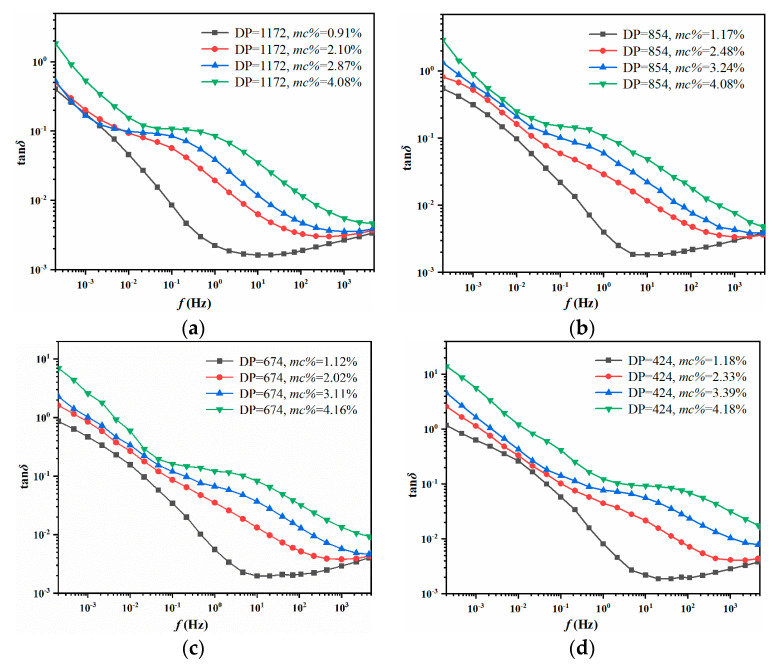

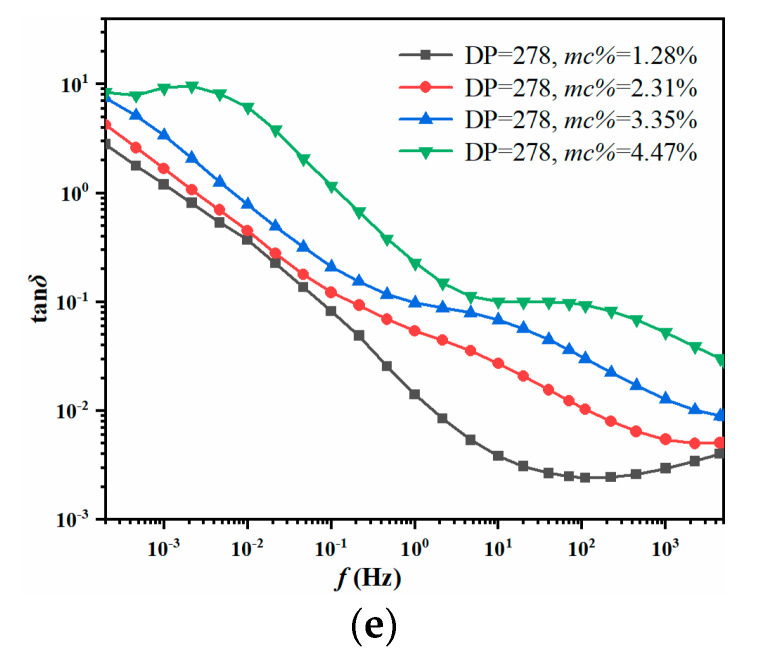
Tan*δ* curves of prepared samples. (**a**) Aged 0 day; (**b**) Aged 1 day; (**c**) Aged 3 days; (**d**) Aged 7 days; and (**e**) Aged 14 days.

The dielectric response experiment is executed at 45 °C, where the test voltage is AC 200V and the test frequency section is 2 × 10^−4^ Hz–5000 Hz. In this case, the tan*δ* curves of oil-immersed pressboards with various moisture content and aging degrees can be seen in Figure 2.

The previous studies revealed that the aging effect will alter the response curves in the low-frequency regions, while the curves in the high-frequency part will almost not be affected. On the contrary, the observed moisture effect always makes the response curve changed in the entire frequency regions. Therefore, the feature parameters extracted by the response curve in the high-frequency regions are regarded as an available tool for analyzing the moisture effect [26,27]. The integral value of tan*δ* curves can be thus chosen as the feature parameter (fingerprint) to realize the moisture diagnosis.

Therefore, in order to collect more moisture information from tan*δ* curves, as well as avoid the impact of aging, the integral values in three characteristic ranges of the middle-high frequency sections of tan*δ* curves are selected as the dielectric fingerprints (*D*_1_–*D*_3_) to predict the moisture inside the oil-immersed insulation, as shown in Equation (1):

(1) $$\{\begin{array}{c} D_{1}=\int_{10^{-1}}^{10^{1}}tan\delta \left(f\right)df\times 10^{2}\\ D_{2}=\int_{10^{2}}^{10^{3}}tan\delta \left(f\right)df\times 10^{1}\\ D_{3}=\int_{10^{3}}^{5\times 10^{3}}tan\delta \left(f\right)df\times 10^{0} \end{array}$$

where *D_i_* (*i* = 1, 2, 3) represents the dielectric fingerprints the and the multiplied coefficient is to keep *D_i_* in a similar data dimension [24]. In addition, the contribution of moisture effect and the aging effect on tan*δ* curves is too similar to identify. Besides, the different combinations of moisture and aging may lead to a similar shape of tan*δ* curves [11,24,26]. In this case, the evaluation results of both aging and moisture are unreliable. In view of this issue, the DC conductivity of insulating oil (*σ_oil_*) is introduced as an auxiliary fingerprint (*D*_4_). If the value of *D*_4_ is relatively large, the shape of tan*δ* curve is dominated by the moisture effect due to the fact that moisture is the leading factor of *σ_oil_*. On the contrary, the shape of the tan*δ* curve is greatly affected by the aging effect when the value of *D*_4_ is relatively small. Moreover, observe the oil-immersed sample with the same aging status (*DP* value) shown in Table 3; the *σ_oil_* changes greatly (633, 2200, 1359, 1375, and 1667 times, respectively) with the moisture increase to 4% from 1%. In summary, when the measured FDS curves convey a similar shape, the *σ_oil_* could be utilized as an available tool for distinguishing the moisture effect, such that the property could especially promote the moisture diagnosis.

(2) $$D_{4}=\sigma_{oil}$$

Afterwards, the integral values in Figure 2 are extracted based on Equation (1) and the DC conductivity is measured by a DIRANA tester. Above all, the fingerprints *D*_1_–*D*_4_ of the prepared oil-immersed pressboards can be acquired and are shown in Table 3.

### 3.2. Construction of Fitting Analysis Model

**Figure 3:**
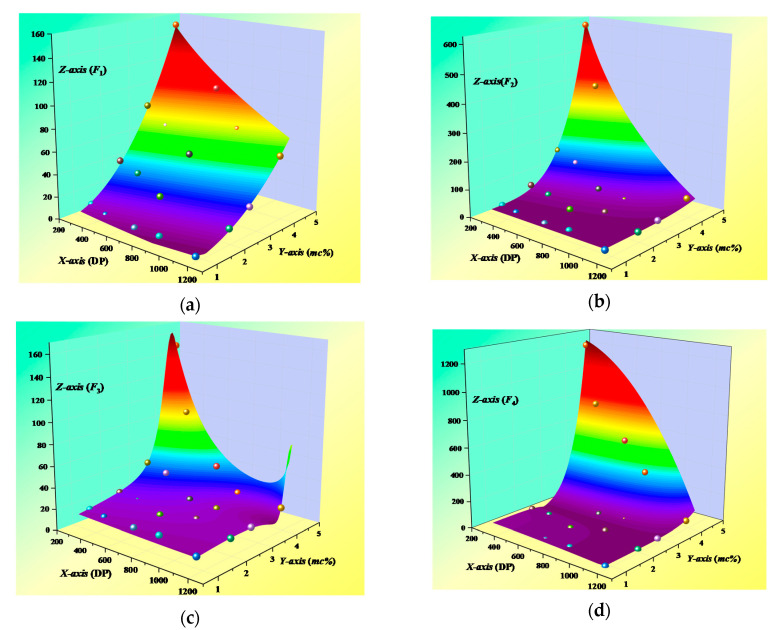
Surface of the fitting analysis model. (**a**) Fitting surface of *F*_1_; (**b**) Fitting surface of *F*_2_; (**c**) Fitting surface of *F*_3_; and (**d**) Fitting surface of *F*_4_.

In part A, 20 groups of dielectric fingerprints have been extracted, which are aimed at reflecting the moisture level of oil-immersed insulation. However, if only 20 groups of dielectric fingerprints are utilized to carry out the training of the GA-SVM multi-classifier, it will give rise to the underfitting of the model, which restricts its learning ability. While the approach for collecting the adequate dielectric fingerprints is theoretically available by preparing a great number of oil-immersed pressboards with various *mc%* and *DP*. However, it will be an extremely heavy work in actual operation due to the preparation duration, materials, and accuracy. Considering this situation, a fitting analysis model is proposed in this work to obtain sufficient fitting fingerprints. Then, the fitting analysis model will be utilized to calculate the fitting fingerprints (*F*_1_–*F*_4_) to construct the training set. The construction of the fitting analysis model is carried out as the following steps:

- The fitting fingerprints *F_i_* is defined as the dependent variable *Z_i_*, *i* = 1, 2, 3, 4, which is set to represent the integral value of tan*δ* curves. Moreover, the value of *DP* and *mc%* are defined as independent variable *X* and *Y*, respectively, which make an obvious impact on the shape of tan*δ* curves;
- Determining the types of fitting functions to build the model with the higher goodness of fitting. Two types of functions (Power 2D and Rational Taylor) are selected to realize the construction of the fitting analysis model in this work. Moreover, the values of *F_i_* cannot be negative, the fitting functions are thus added with an absolute operation;
- The 20 groups of original data shown in Table 3 are brought into the determined functions so the fitting analysis model is constructed;
- Adjust the parameters of the model. The values of parameters can largely determine the goodness of the model. After experiments and adjustments, the parameters of the model are decided and the fitting analysis model is established. Finally, the goodness of *F_i_* (*i* = 1–4) reach to 0.988, 0.983, 0.999, and 0.989, respectively. The surface of the fitting analysis model is shown in Figure 3, and its parameters and formulates are displayed in Table 4.

## 4. Construction of the GA-SVM Moisture Content Prediction Model

The introduction of SVM and GA is given in this chapter. Combining the training samples provided by the reported fitting analysis model, the GA-SVM model utilized for moisture prediction is later constructed.

### 4.1. The Introduction of SVM

The support vector machine is developed based on the statistical learning theory [28]. It uses the principle of structural risk minimization to increase the generalization capability of the classification model [29]. The sample points are mapped into the high-dimensional space with the help of kernel function to realize linearly separable, which are linearly inseparable in the low-dimensional space. Then, the optimal hyperplane is established to accomplish the classification of samples. An ordinary hyperplane can be expressed by Equation (3).

(3) $$\omega^{T}x+b=0$$

If the sample points (*x_i_*, *y_i_*) meet the condition expressed in Equation (4), the classification result is correct.

(4) $$y_{i}\left(\omega^{T}x_{i}+b\right)\geq 1,i=1,2,\ldots ,l$$

If the classification margin of the two types of sample data reaches the maximum, the hyperplane in Equation (3) will become the optimal hyperplane, which can be expressed by Equation (5).

(5) Φmin(ω,ξ)=12‖ω‖2+C∑i=1lξis.t.yi[ωTϕ(xi)+b]≥1−ξis.t.ξi≥0 , i=1,2,…,l

where, the *ξ_i_* is called the slacked variable, and the introduction of *ξ_i_* allows the existence of the misclassified samples. The *C* is a penalty factor, and the value of *C* reflects the importance attached to misclassified samples. Besides, ϕ(⋅) maps the sample points from the original space to the feature space. By using the Lagrange optimization method to solve the quadratic optimization problem in (5), Equation (6) will be later gotten.

(6) $$L\left(\omega ,\xi ,b,\alpha ,\beta \right)=\Phi (\omega ,\xi )-\sum_{i=1}^{l}\alpha_{i}\left\{y_{i}\left[\omega^{T}\phi (x_{i})+b\right]-1+\xi_{i}\right\}-\sum_{i=1}^{l}\beta_{i}\xi_{i}$$

Where *α_i_* and *β_i_* are called the Lagrange operator. If Equation (6) satisfies the conditions obtained by Lagrange vertical multiplication shown in Equation (7), Equation (5) can be converted to the dual form shown in Equation (8).

(7) $$\left\{ \begin{array}{l} \frac{\partial L}{\partial \omega }=0\Rightarrow \omega =\sum_{i=1}^{l}\alpha_{i}y_{i}x_{i}\\ \frac{\partial L}{\partial b}=0\Rightarrow \sum_{i=1}^{l}\alpha_{i}y_{i}=0\\ \frac{\partial L}{\partial \xi_{i}}=0\Rightarrow C-\alpha_{i}-\beta_{i}=0 \end{array}\right.$$

(8) ψmax(α)=∑i=1lαi−12∑i,j=1lαiαjyiyjϕ(xi)⋅ϕ(xj)s.t. 0≤αi≤Cs.t. ∑i=1lαiyi=0

Then, the kernel function is pulled in to simplify the calculations, and the Gaussian radial basis function is selected as a kernel function in this paper, which can be expressed in the form of Equation (9).

(9) $$K(x_{i},x_{j})=\exp(-\gamma {\left\|x_{i}-x_{j}\right\|}^{2})\;,\;\gamma >0$$

Where, *γ* is the key parameter of the Gaussian radial basis kernel function. The decision function brought in the kernel function can be shown in Equation (10).

(10) $$f(x)=sign[\sum_{i=1}^{l}\alpha_{i}y_{i}K(x,x_{i})+b]$$

### 4.2. The Introduction of GA

**Figure 4:**
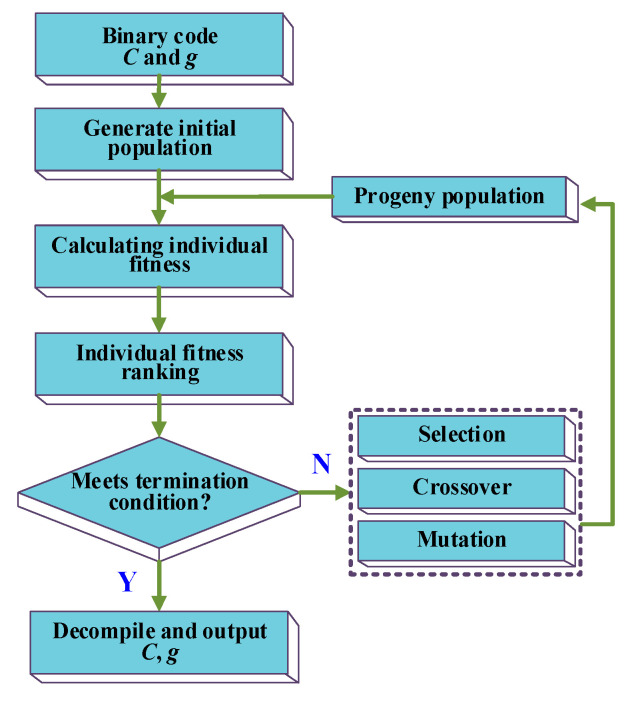
Optimizing process of genetic algorithm.

The parameters *C* and *g* (*g* = *γ*) reported in part A can affect the performance of the SVM model, and the genetic algorithm can optimize the performance of SVM by adjusting the values of *C* and *g*, where the optimizing process is shown in Figure 4.

The parameters optimization by using the genetic algorithm can be realized by employing the following steps:

- *C* and *g* should be firstly defined, and then operate binary-coded.
- Generating an initial population containing *M* individuals, where *M* is the number of individuals in the initial population.
- Calculating the fitness of individuals in the population by Equation (11):
  (11) $$fitness=\frac{N_{T}}{N_{T}+N_{F}}$$
  where, *N_T_* and *N_F_* represent the number of correctly classified and incorrectly classified samples, respectively.
- Ranking the individuals based on the fitness, then judging whether the termination condition meets the required level; if so, end the iteration, if not, go to step V.
- Generating the progeny by genetic operators. Then, go to step III.

The genetic operator in the above process consists of selection, crossover, and mutation. Among them, the selection is realized by the method of roulette wheel selection. In this method, individuals with higher fitness will occupy a larger area in the roulette so that they are more likely to be selected. Besides, crossover and mutation are the main and secondary ways to produce the offspring different from the parents to enhance the diversity of the population.

In the loop of Figure 4, the genetic algorithm continuously adjusts the constituent of the parameter population based on its fitness, and removes the individuals with a lower level of fitness. As the number of iterations increases, the increasing number of high-quality individuals consists of the new population. Finally, the parameter optimization is completed the and the optimized parameters are outputted. 

### 4.3. Prediction Model of the Moisture Content Based on GA-SVM

#### 4.3.1. Construction of Sample Set

The training set is obtained by the reported fitting analysis model shown in Figure 3. The sample’s data is obtained by taking points at equal steps from the surface (a)–(d) presented in Figure 3. It can be learned from Table 2 that the value range of *DP* is from 279 to 1172 and the value range of *mc%* is from 0.91 to 4.47. 

Therefore, this study presets the available value ranges of *mc%* (1%–4.5%) and *DP* (280–1170). The step size of *DP* value and *mc%* is preset to 10 and 0.5%, respectively. Then, a total of 720 sample points are obtained. Afterwards, mapping the sample points into the surfaces in Figure 3, and 720 sets of fitting fingerprints (*F_i_*) corresponding to different *mc%* and *DP* values are obtained. Lastly, the 720 sets of fitting fingerprints are used to finish the construction of the training set of GA-SVM model.

#### 4.3.2. Model Building and Parameter Optimization

The reported 720 sets of fitting fingerprints are employed to accomplish the training of the GA-SVM model and the genetic algorithm is utilized to optimize the parameters *C* and *g*. 

As shown in Step I (Section 4.2), the variation range of the key parameters *C* and *g* are firstly determined, as shown in Equation (12):

(12) $$\left\{ \begin{array}{l} \left[C_{\min},C_{\max}\right]=\left[0,100\right]\\ \left[g_{\min},g_{\max}\right]=\left[0,1000\right] \end{array}\right.$$

**Figure 5:**
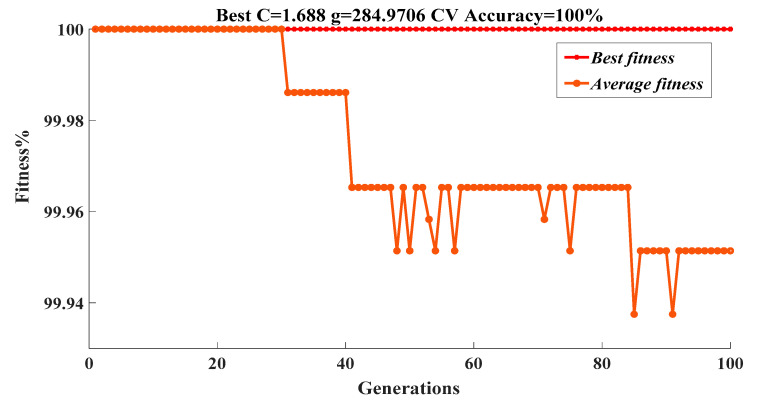
Optimization of parameters by genetic algorithm.

Then, according to Step II, the number *M* of individuals in the initial population is determined, *M* = 20. And 10-fold cross-validation is selected to obtain the cross-validation accuracy of the GA-SVM model. Moreover, considering the accuracy of cross-validation and the time cost of training, 200 is selected as the maximum value of iterations in this work. After completing the above series of settings, the following Steps (III, IV, V) are carried out using the Matlab program. The optimization process of parameter *C* and *g* is shown in Figure 5. 

The optimization is terminated when the number of iterations reaches 100, it is because the cross-validation accuracy of the GA-SVM model is close to 100% (Much larger than 95%). As a result, the optimized global optimal parameters are output with the best *C* = 1.688 and the best *g* = 284.9706. 

In addition, it can be learned from Figure 5 that the best fitness of the generations is stable at 100% and the average fitness fluctuates between 100% and 99.93%. Moreover, the cross-validation accuracy of the GA-SVM model on the training set reaches 100%. In this way, the construction of the GA-SVM model is achieved.

#### 4.3.3. Controlled Trial

**Figure 6:**
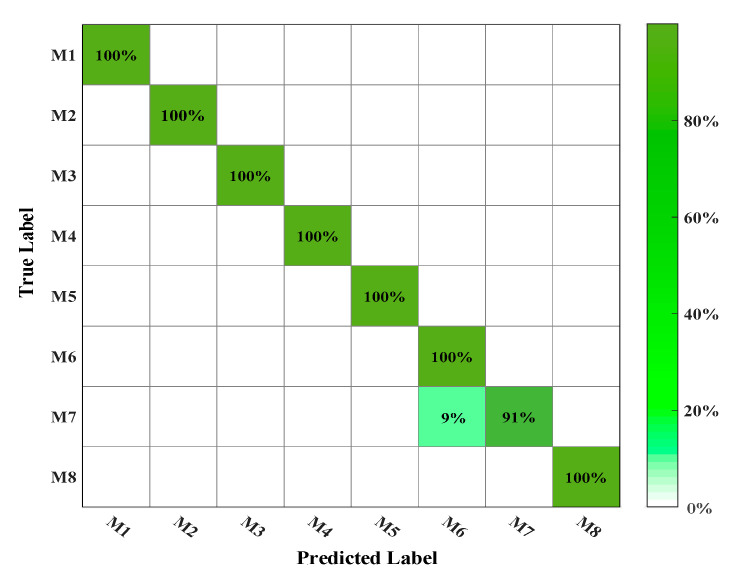
Classification result of the support vector machine (SVM).

As a controlled trial, the MATLAB R2019b software and the same 720 training sets are utilized to train the SVM model (parameters are not optimized by the genetic algorithm). The 10-fold cross-validation and Gaussian radial basis functions are also applied during the training. The classification result of the SVM model on the training sets is shown in Figure 6.

The classification results indicate that the CV accuracy of the SVM model is 98.9% and 9% of the training samples that should be classified into M7 are misclassified into M6. Above all, it can be learned from the controlled trial that the application of the genetic algorithm can improve the classification accuracy of the SVM model.

## 5. Feasibility Verification of the GA-SVM Model for Moisture Prediction

After accomplishing the construction of the GA-SVM moisture diagnosis model, the verification experiments are performed to prove the feasibility and accuracy of the moisture diagnosis model in lab and field conditions.

### 5.1. Verification under Laboratory Conditions

**Figure 7:**
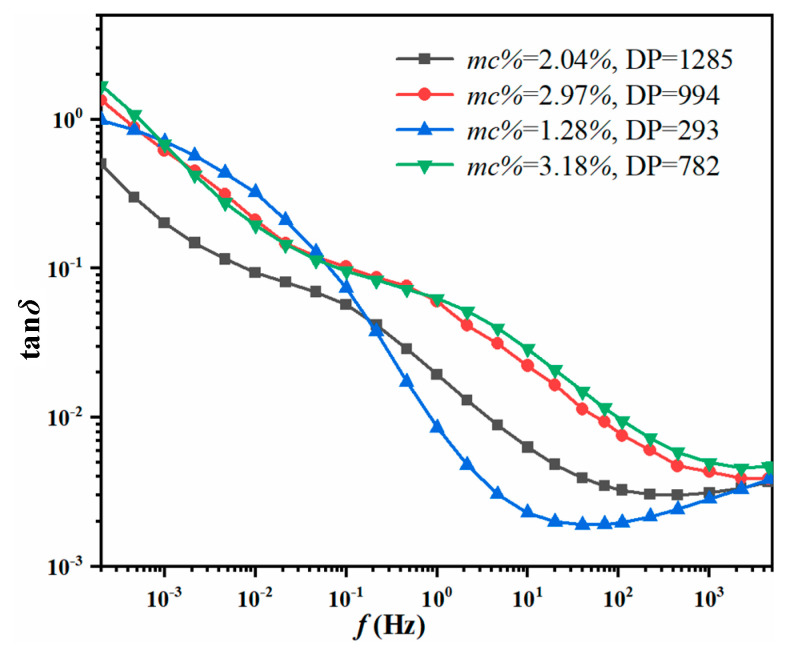
Tan*δ* curves of the lab samples.

Two types of cellulosic pressboards (as shown in Table 5) and Karamay No.25 naphthenic mineral oil are utilized to prepare the testing oil-immersed pressboards. The purpose of this experimental design is to discuss whether the reported GA-SVM model could be applied to the cellulose materials with different thicknesses. Then, four groups of testing samples with different *mc%* and *DP* are prepared and the FDS curves are tested according to the steps shown in Figure 1. The insulating information of the testing lab samples is tested by Karl Fischer titrator and viscosity tester, which is shown in Table 6. Moreover, the tan*δ* curves of the lab samples can be seen in Figure 7.

For the feasibility verification of the reported GA-SVM model, the dielectric fingerprints of the lab samples are extracted by Equation (1). Combining the tested *σ_oil_*, the dielectric fingerprints of lab samples can be collected, which are shown in Table 7.

**Figure 8:**
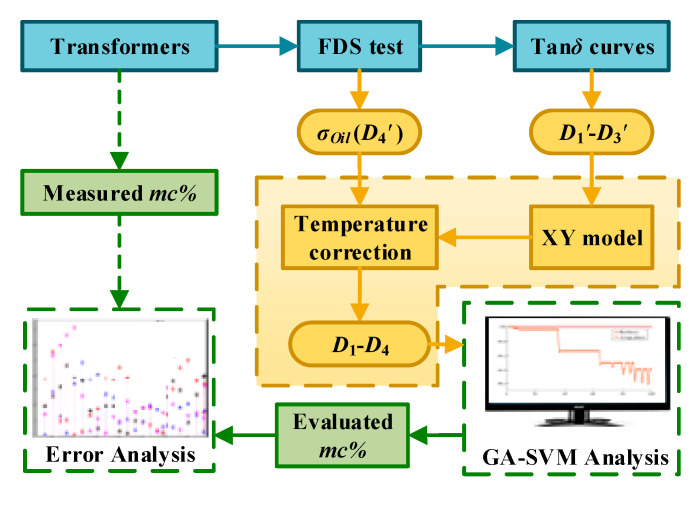
Tan*δ* curves of the lab samples.

The four groups of dielectric fingerprints in Table 7 are input to the reported GA-SVM model, and the moisture diagnosis results can be obtained, as shown in Figure 8, where the percentage error (*P.E)* in Table 8 is computed by Equation (13).

(13) $$P.E=\left|\frac{\mathrm{Predicted}\;mc\%-\mathrm{Measured}\;mc\%}{\mathrm{Measured}\;mc\%}\right|\times 100\%$$

As shown in Table 8, the measured *mc%* of lab samples is 2.04%, 2.97%, 1.28%, and 3.18%, and it is classified as M3, M5, M2, and M5, respectively. As a result, the reported GA-SVM moisture prediction model achieves the correct classification of the lab samples and the percentage error is 1.96%, 1.01%, 17.19%, and 5.66%.

From the above experiments, the calculated average percentage error of lab testing samples is 6.46%, so the reported GA-SVM model realized a relatively reliable moisture prediction for the lab samples.

### 5.2. Verification under Field Conditions

The three-winding transformers with different loading histories under maintenance are selected in this part to verify the field application of GA-SVM. The details of them are shown in Table 9.

The DIRANA is used to the FDS test of the main insulation system between the high and medium voltage windings, as well as the inside moisture. The scheme of the assessment activities is given in Figure 8, where *D*_1_–*D*_4_ are the original measured values and *D*_1_′–*D*_4_′ is the dielectric fingerprints processed by the XY model and temperature correction.

**Figure 9:**
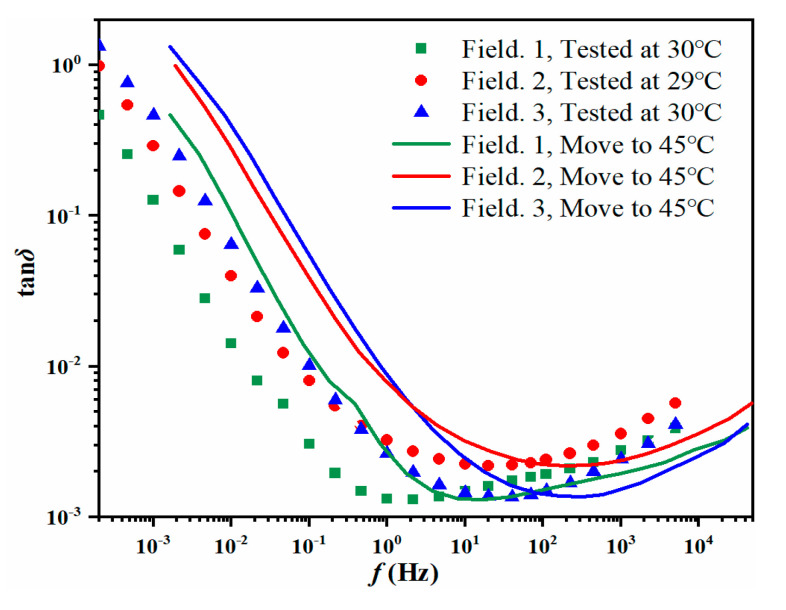
Temperature correction of field data.

The field verification can be achieved by referring to the following steps:

- Testing the complex capacitance C_tot_*(ω) of the above field transformers. The complex dielectric constant ε_tot_*(ω) is calculated by the formula ε_tot_*(ω) = C_tot_*(ω)/*C*_0_.
- In this work, the structure parameters *X* and *Y* of the tested transformers are obtained by the commercial dielectric response analyzer, i.e., DIRANA/OMICRON. The DIRANA could access the preset curve that is most similar to the measured curve by using match technique. Then, the obtained structure parameters (*X* and *Y*) of the preset curve is approximatively regarded as the tested sample’s parameters. The analysis results of *X* and *Y* are shown in Table 10.
- The XY model is utilized to calculate the ε_PB_*(ω) of transformer oil-immersed pressboards, as shown in Equation (14).
  (14) $$\left\{ \begin{array}{l} \varepsilon_{tot}^{*}(\omega )=\frac{1-Y}{\left(1-X)/\varepsilon_{oil}^{*}(\omega )+X/\varepsilon_{PB}^{*}(\omega \right)}+Y\times \varepsilon_{PB}^{*}(\omega )\\ \varepsilon_{oil}^{*}(\omega )=2.2-j\cdot \sigma (T)/(\varepsilon_{0}\omega ) \end{array}\right.$$
- Correcting the influence caused by various test temperatures. The FDS data of field transformers is measured at 30 °C, 29 °C, and 30 °C, respectively, while the data of the reported GA-SVM model is measured at 45 °C. Therefore, the shift factor αT shown in Equation (15) is applied to realize the temperature correction of the FDS data at different test temperatures.
  (15) $$\alpha_{T}=\mathrm{EXP}[\frac{E_{a}}{R}(\frac{1}{T}-\frac{1}{T_{ref}})]$$
  where *E_a_* represents the activation energy (*E_a_* =113kJ/mol). *R* represents a gas constant and *R* = 8.314 J/(mol·K). *T_ref_* represents the reference temperature (*T_ref_* = 318.15 K). *T* represents the test temperature (*T*_1_ = 303.15 K; *T*_2_ = 302.15 K; *T*_3_ = 303.15 K). Then, the shift factor *α_T_*_1_, *α_T_*_2_, *α_T_*_3_ is calculated (*α_T_*_1_ = 8.28; *α_T_*_2_ = 9.60; *α_T_*_3_ = 8.28). Above all, the temperature correction of the measured FDS data is realized. The corrected tan*δ* curves are shown in Figure 9, where the scattered points are uncorrected data, and the curves are corrected by the shift factor. The dielectric fingerprints *D*_1_*–D*_3_ can be extracted from these correct curves by using Equation (1). Besides, it has been proved in studies [24,30] that the temperature correction of *σ_oil_* can be achieved by multiplying the shift factor *α_T_*, in that way, *D*_4_ = *σ_oil_* × *α_T_*. Above all, the dielectric fingerprints are obtained and shown in Table 11.
- Get the dielectric fingerprints *D*_1_–*D*_4_ inputted to the reported GA-SVM model. The *mc*% prediction of the field transformers is realized, and the results are shown in Table 12. The prediction *mc*% in Table 12 is from GA-SVM and the measured *mc*% is from DIRANA. It can be seen that the same moisture level is given for Field 1 and Field 3. As for Field 2, the number of operating years is 8. Considering that the normal operating life of the transformer is 30 years, the Field 2 transformer is still lying in the early-term stage. Therefore, the predicted results of DIRANA and GA-SVM are both within a reasonable range.

In summary, the feasibility and accuracy of the reported GA-SVM model have been preliminarily proved based on the laboratory and field data shown in Table 8 and Table 12, respectively.

## 6. Conclusions

Compared with the traditional moisture predicted method. A novel idea is proposed in this paper, which constructs the quantitative relationship between FDS feature parameters and the moisture. In that way, the fitting analysis model is employed to expand the number of training samples so that the GA-SVM can be trained and further applied to accomplish the moisture prediction of transformer oil-immersed polymer insulation. The analysis and findings can be summarized as follows:

- A group of dielectric fingerprints *D*_1_–*D*_4_, sensitive to the moisture content inside the transformer oil-immersed insulation, can be obtained by the presented analysis.
- A small number of samples are difficult to realize the training of the SVM multi-classifier, so a fitting analysis model is proposed in this paper to expand the number of training samples. Consequently, the heavy work of preparing a great number of oil-immersed pressboards is replaced and the training set with adequate fitting fingerprints is obtained easily.
- In the course of the construction of the moisture prediction model, the genetic algorithm is utilized to optimize the key parameters of the SVM. It has been proved that the GA-SVM performs better than the SVM on the classification of the training set.
- The feasibility and accuracy of the moisture prediction model based on the GA-SVM are proved by the laboratory and field experiments. The reported GA-SVM achieves the correct classification of the moisture levels of the lab samples, and the average relative error of the lab sample is 6.46%. As for the field transformer, considering the measured results of DIRANA and the operating years, the predicted values of GA-SVM is within a reasonable range.

## Figures and Tables

**Table 1:** Details of used cellulosic pressboard and insulating oil.

Cellulosic Pressboard		Insulating Oil	
Brand	T_4_ transformer pressboard	Brand	Karamay No.25 naphthenic mineral oil
Thickness	1.05 mm	Tan *δ*	4 × 10^−4^
Tensile strength	Machine Direction: 138.8 MPa, Cross-Machine Direction: 97.1 MPa	Pour point	≤−45 °C
Density	1.09 g/cm^3^	Flash point	135 °C

**Table 2:** Classification of moisture content in oil-immersed insulation.

*mc%*	1.0%	1.5%	2.0%	2.5%	3.0%	3.5%	4.0%	4.5%
Tag	M1	M2	M3	M4	M5	M6	M7	M8
Damp State	Well Dry	Dry	Slightly Damped	Damped	Moderately Damped	Quiet Damped	Severely Damped	Extremely Damped

**Table 3:** Dielectric fingerprints (*D*_1_–*D*_4_) of prepared oil-immersed pressboards.

No.	Insulation Information		Dielectric Fingerprint			
	*DP*	*mc%*	*D*_1_	*D*_2_	*D*_3_	*D*_4_
1	About 1172	0.91	1.88	21.30	12.47	0.06
2		2.10	11.41	27.32	13.84	3.50
3		2.87	22.00	33.55	14.87	11.00
4		4.08	55.24	62.62	19.50	38.00
5	About 854	1.17	2.67	23.89	14.36	0.15
6		2.48	18.55	32.79	14.06	5.80
7		3.24	35.98	44.83	15.85	19.00
8		4.08	70.53	91.23	22.51	330.00
9	About 674	1.12	3.47	22.70	14.44	0.39
10		2.02	22.03	35.72	16.23	7.80
11		3.11	50.17	67.23	19.53	25.00
12		4.16	102.56	163.36	42.06	530.00
13	About 424	1.18	4.76	22.20	13.79	0.56
14		2.33	31.72	41.80	16.88	9.70
15		3.39	67.24	124.22	34.23	31.00
16		4.18	105.98	392.64	88.70	770.00
17	About 279	1.28	8.25	23.82	14.47	0.72
18		2.31	39.01	59.43	20.42	17.00
19		3.35	82.62	156.31	40.30	56.00
20		4.47	152.11	604.64	149.71	1200.00

**Table 4:** Formula and parameters of the fitting analysis model.

**Equation:**	$Z_{1}=\left|A_{0}+A_{1}\cdot pow\left(X,A_{2}\right)+A_{3}\cdot pow\left(Y,A_{4}\right)+A_{5}\cdot pow\left(X,A_{2}\right)\cdot pow\left(Y,A_{4}\right)\right|$				
*A*_0_	6.759	A3	8.401	Precision	10^−6^
*A*_1_	−0.418	*A*_4_	2.217	R-Square	0.988
*A*_2_	0.462	*A*_5_	−0.221		
**Equation:**	$Z_{2}=\left|A_{0}+A_{1}\cdot pow\left(X,A_{2}\right)+A_{3}\cdot pow\left(Y,A_{4}\right)+A_{5}\cdot pow\left(X,A_{2}\right)\cdot pow\left(Y,A_{4}\right)\right|$				
*A*_0_	26.39	*A*_3_	−78.58	Precision	10^−6^
*A*_1_	−2.092	*A*_4_	4.910	R-Square	0.983
*A*_2_	−0.0033	*A*_5_	80.42		
**Equation:**	$Z_{3}=\left|\frac{A_{0}+A_{1}\cdot X+A_{2}\cdot Y+A_{3}\cdot Y^{2}+A_{4}\cdot X\cdot Y}{1+A_{5}\cdot X+A_{6}\cdot Y+A_{7}\cdot X^{2}+A_{8}\cdot Y^{2}+A_{9}\cdot X\cdot Y}\right|$				
*A*_0_	10.57	*A*_5_	−3.3 × 10^−5^	Precision	10^−6^
*A*_1_	−0.0019	*A*_6_	1.7 × 10^−7^		
*A*_2_	−0.519	*A*_7_	−1.2 × 10^−10^	R-Square	0.999
*A*_3_	−1.337	*A*_8_	−0.467		
*A*_4_	0.238	*A*_9_	0.055		
**Equation:**	$Z_{4}=\left|A_{0}+A_{1}\cdot pow\left(X,A_{2}\right)+A_{3}\cdot pow\left(Y,A_{4}\right)+A_{5}\cdot pow\left(X,A_{2}\right)\cdot pow\left(Y,A_{4}\right)\right|$				
*A*_0_	−28.50	*A*_3_	0.0098	Precision	10^−6^
*A*_1_	4.6 × 10^−6^	*A*_4_	7.888	R-Square	0.989
*A*_2_	2.229	*A*_5_	−1.3 × 10^−9^		

**Table 5:** Details of two types of cellulosic pressboards.

Pressboard I		Pressboard II	
Brand	T4 pressboard	Brand	Common pressboard
Thickness	1 mm	Thickness	2 mm
Tensile strength	Machine Direction: 138.8 MPa, Cross-Machine Direction: 97.1 MPa	Tensile strength	Machine Direction:150.0 MPa, Cross-Machine Direction: 57.1 MPa
Density	1.09 g/cm^3^	Density	1.17 g/cm^3^

**Table 6:** Insulating information of the lab samples.

Testing Samples	*mc%*	DP
Lab. 1	2.04%	1285
Lab. 2	2.97%	994
Lab. 3	1.28%	293
Lab. 4	3.18%	782

**Table 7:** Dielectric fingerprints of the lab samples.

No.	Dielectric Fingerprints			
	*D*_1_	*D*_2_	*D*_3_	*D*_4_
Lab. 1	12.04	27.32	13.84	0.58
Lab. 2	37.12	44.83	15.85	6.62
Lab. 3	5.20	22.00	13.84	1.10
Lab. 4	42.93	53.93	18.74	76.00

**Table 8:** Prediction results of the lab samples.

No.	Predicted *mc%*	Measured *mc%*	T/F	*P. E*
Lab. 1	2.0%(M3)	2.04%(M3)	T	1.96%
Lab. 2	3.0%(M5)	2.97%(M5)	T	1.01%
Lab. 3	1.5%(M2)	1.28%(M2)	T	17.19%
Lab. 4	3.0%(M5)	3.18%(M5)	T	5.66%

**Table 9:** Details of field transformers.

No.	Voltage Level	Test Voltage	Test Temperature	*σ* (T)	Service Record	
Field 1	110 kV	200 V	30 °C	2.1 pS/m	1 years
Field 2	110 kV	200 V	29 °C	6.3 pS/m	8 years
Field 3	220 kV	200 V	30 °C	220 pS/m	14 years

**Table 10:** Structure parameters of the main insulation system.

No.	*X*	*Y*
Field 1	0.27	0.20
Field 2	0.29	0.24
Field 3	0.30	0.14

**Table 11:** Dielectric fingerprints of field transformers.

No.	Dielectric Fingerprints			
	*D*_1_	*D*_2_	*D*_3_	*D*_4_
Field 1	1.65	14.64	5.93	17.33
Field 2	5.01	21.77	8.75	60.35
Field 3	4.81	11.63	4.89	181.5

**Table 12:** Prediction results of field transformers.

No.	Predicted *mc%*	Measured *mc%*
Field 1	1.0%(M1)	0.7%(M1)
Field 2	1.5%(M2)	1.2%(M1)
Field 3	1.0%(M1)	0.8%(M1)
